# Biodegradable nanoparticles combining cancer cell targeting and anti-angiogenic activity for synergistic chemotherapy in epithelial cancer

**DOI:** 10.1007/s13346-021-01090-6

**Published:** 2022-01-01

**Authors:** Francesca Moret, Claudia Conte, Diletta Esposito, Giovanni Dal Poggetto, Concetta Avitabile, Francesca Ungaro, Natascia Tiso, Alessandra Romanelli, Paola Laurienzo, Elena Reddi, Fabiana Quaglia

**Affiliations:** 1 Department of Biology, University of Padova, Padova, 35121 Italy; 2 Department of Pharmacy, University of Napoli Federico II, Napoli, 80131 Italy; 3 Institute for Polymers, Composites and Biomaterials, CNR, Pozzuoli, 80078 Italy; 4 Institute of Biostructure and Bioimaging, CNR, Napoli, 80134 Italy; 5 Department of Pharmaceutical Sciences, University of Milan, Milano, 20133 Italy

**Keywords:** Polymeric nanoparticles, Anti-angiogenic peptides, Folate targeting, Tumor spheroids, Xenografted zebrafish embryos

## Abstract

A biodegradable engineered nanoplatform combining anti-angiogenic activity and targeting of cancer cells to improve the anticancer activity of docetaxel (DTX) is here proposed. Indeed, we have developed biodegradable nanoparticles (NPs) of poly(ethylene glycol)-poly(ε-caprolactone), exposing on the surface both folate motifs (Fol) for recognition in cells overexpressing Folate receptor-α (FRα) and the anti-angiogenic hexapeptide aFLT1. NPs showed a size around 100 nm, the exposure of 60% of Fol moieties on the surface, and the ability to entrap DTX and sustain its release with time. NPs were stable in simulated biological fluids and slightly interacted with Fetal Bovine serum, especially in the formulation decorated with Fol and aFLT1. The presence of Fol on NPs did not impair the anti-angiogenic activity of aFLT1, as assessed by in vitro tube formation assay in HUVEC endothelial cells. In both 2D and 3D KB cell cultures in vitro, the cytotoxicity of DTX loaded in NPs was not significantly affected by Fol/aFLT1 double decoration compared to free DTX. Remarkably, NPs distributed differently in 3D multicellular spheroids of FRα-positive KB cancer cells depending on the type of ligand displayed on the surface. In particular, NPs unmodified on the surface were randomly distributed in the spheroid, whereas the presence of Fol promoted the accumulation in the outer rims of the spheroid. Finally, NPs with Fol and aFLT1 gave a uniform distribution throughout the spheroid structure. When tested in zebrafish embryos xenografted with KB cells, NPs displaying Fol/aFLT1 reduced DTX systemic toxicity and inhibited the growth of the tumor mass and associated vasculature synergistically. Overall, nanotechnology offers excellent ground for combining therapeutic concepts in cancer, paving the way to novel multifunctional nanopharmaceuticals decorated with bioactive elements that can significantly improve therapeutic outcomes.

**Graphical abstract:**

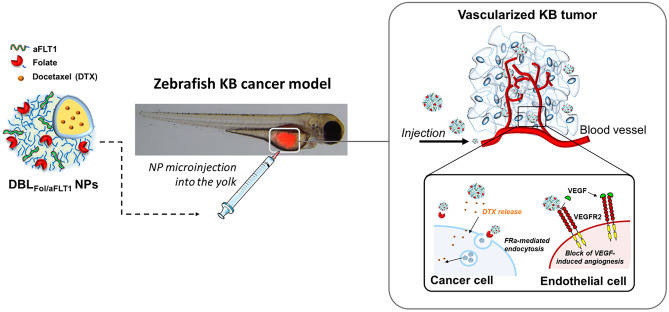

**Supplementary information:**

The online version contains supplementary material available at 10.1007/s13346-021-01090-6.

## Introduction

In the vast arena of nanoplatforms developed so far for the delivery of chemotherapeutics, multifunctional polymeric nanoparticles (NPs) bearing different elements in a single entity offer an unprecedented tool of innovation [1–4]. This type of NPs can be engineered: (i) to transport the chemotherapeutic in the desired pathological area; (ii) to target specific cell population in the tumor microenvironment; (iii) to deliver multiple drugs and attain synergic/additive effects.

Amphiphilic diblock copolymers of poly(ethylene glycol) and polyesters are biocompatible and biodegradable versatile materials that form a wide range of core–shell nanostructures and deliver multiple drugs in a sustained manner [5–7]. The presence of an outer PEG fringe makes the surface hydrophilic, which is helpful in limiting some undesired interactions with the biophase (opsonization in the blood compartment) [8] and facilitating transport through mucosal tissues or extracellular matrix [9–11]. PEGylation is a two swords strategy since the benefits mentioned above are coupled with a poor attitude of NPs to cross the cell membrane and deliver drug cargo inside cells [12]. For these reasons, surface-modification of NPs with ligands binding cell membrane receptors has been attempted to improve their selective internalization in specific cell types of tumor microsystem [3, 13].

Folate receptor-α (FRα) is one of the most studied surface receptors employed for this purpose since it is overexpressed in several cancer types [14]. However, the control over Folate (Fol) exposure on NPs surface is not trivial at all when assembling NPs from all in one block copolymers [15] and poorly investigated [13, 16]. We have recently proposed different strategies for the effective display of Fol motifs on the surface of PEGylated NPs through appropriate tailoring of polymer block lengths and fabrication conditions [16–18]. Results have demonstrated a certain degree of specificity of the NPs for cancer cells overexpressing FRα, although no gain in cytotoxicity was observed.

Besides being a somewhat common targeting strategy, surface modification of NPs with functional motifs represents a unique tool to impart synergistic effects [2]. In the context of cancer, angiogenesis is an orchestrated process crucial for the growth, invasion, and metastasis of a primary tumor [19]. The concept that dysfunctional vasculature is common to a wide range of solid cancers has pushed the interest in tumor vascular targeting [20, 21]. Furthermore, stromal cells, unlike neoplastic cells, are genetically more stable, being less prone to develop resistance to therapy [22]. VEGF is the most relevant player in tumor angiogenesis since its inhibition influences endothelial cell survival/growth/migration, blood flow, and stromal cell recruitment [23]. Anti-angiogenic therapy in cancer has reached maturity, and distinct types of angiogenic inhibitors, such as antibodies and small molecules, have been introduced in the clinic (bevacizumab, tyrosine kinase inhibitors) to potentiate the response to chemotherapy, patient outcome, and survival in different cancer types [24]. However, non-selective anti-angiogenic therapies require repeated i.v. administration [25] and may lead to a worse response in terms of drug resistance, invasion, and metastasis [26]. To address the urgent need for new nano-oriented approaches in anti-angiogenic therapies [27], we have recently built up NPs of PEG-poly(ε-caprolactone) (PEG-PCL) surface-modified with the anti-angiogenic anti-FLT1 hexapeptide (aFLT1, GNQWFI), which selectively binds VEGFR1 isoform expressed in tumor dysfunctional capillaries [28, 29]. We found that NPs decorated with aFLT1 were superior to free aFLT1 in inhibiting tube vessel formation in vitro and potentiated the anticancer activity of docetaxel (DTX) in chicken embryo chorioallantoic membranes (CAM) xenografted with human triple-negative breast cancer cells [30].

Based on these premises, here we propose an improved multifunctional therapeutic NP for the delivery of DTX, which combines aFLT1 anti-angiogenic activity with Fol targeting. NPs, carrying the therapeutic cargo DTX in the core, are prepared from PEG-PCL copolymers conjugated with Fol and aFLT1 at the PEG-OH end group. After an in-depth characterization of NPs, their biological profile was tested in 2D and 3D cultures of FRα over-expressing KB human cervical cancer cells and zebrafish embryos xenografted with the same type of cells.

## Materials and methods

### Materials

mPEG-PCL (mPEG of 1.0 kDa, mPEG-PCL Mn 5.4 kDa), Fol-PEG-PCL (mPEG of 1.0 kDa, PCL Mn 4.7 kDa), and aFLT1-PEG-PCL (mPEG of 1.0 kDa, PCL Mn 4.7 kDa) were synthesized and characterized according to the procedures reported in our previous studies [17]. Docetaxel (DTX) was purchased from LC laboratories (USA). 3,3′-dioctadecyloxacarbocyanine perchlorate (DiO) was purchased from ThermoFisher Scientific (Italy). Sodium chloride, potassium phosphate dibasic and potassium phosphate monobasic, sodium azide, and potassium chloride were used as received from Sigma Aldrich. All other solvents and chemicals were purchased from Sigma Aldrich (Milan, Italy) and used as received.

### Preparation of NPs

We prepared bare PEGylated NPs (DBL) from mPEG-PCL copolymer, folate-targeted NPs (DBL_Fol_) from mPEG-PCL/Fol-PEG-PCL (8:2 by weight, respectively), and anti-angiogenic folate-targeted NPs (DBL_Fol/aFLT1_) from mPEG-PCL/Fol-PEG-PCL/aFLT1-PEG-PCL (7:2:1 by weight, respectively). NPs were obtained by solvent diffusion of an organic phase (10 mg of copolymer/s in 1 mL of acetone) added dropwise in water (2 mL) under magnetic stirring (500 rpm). The addition of a surfactant was not required. After solvent evaporation, NPs were filtered through 0.45 μm Phenex® filters (Phenomenex, USA). NPs loaded with DTX were prepared according to the procedure reported above by adding DTX (1 mg) in acetone. Fluorescent NPs loaded with DiO were prepared with the same method by adding DiO (0.1 mg) in the organic phase.

### NP characterization

The hydrodynamic diameter (D_H_), polydispersity index (PI), and zeta potential (*ξ*) of NPs were determined on a Zetasizer Nano ZS (Malvern Instruments Ltd). Results are reported as the mean of three separate measurements of three different batches ± SD (*n* = 9). The yield of the NPs production process was evaluated on an aliquot of NPs dispersion by weighting the solid residue after freeze-drying. Results are expressed as the ratio of the actual NPs weight to the theoretical polymer or polymer + drug weight × 100 ± SD (*n* = 3).

DTX loading inside NPs was assessed by placing NPs (1 mg) in 500 μL of acetonitrile under stirring and then adding 500 μL of water. After that, the sample was filtered through a 0.45 μm filter (RC, Chemtek, Italy) and analyzed to evaluate the DTX amount according to a previously reported HPLC method [31]. Briefly, the analysis was carried out on a Shimadzu apparatus (Japan) on a Juppiter 5 μm, C18 column. The mobile phase was a 55:45 (v/v) mixture of 0.1% TFA in water and acetonitrile pumped at a flow rate of 1 mL min^−1^. The UV detector was set at 227 nm. A calibration curve for DTX in ethanol was plotted in the concentration range of 2–200 μg mL^−1^. The release of DTX was determined on NPs (0.5 mg) dispersed in 0.5 mL of 10 mM phosphate buffer containing NaCl (137 mM) and KCl (2.7 mM) at pH 7.4 (PBS) at 37 °C and placed in a dialysis bag. The external phase (5 mL) was PBS again. At predetermined times, 1 mL aliquots of the sample were collected and then analyzed by HPLC.

Fixed aqueous layer thickness (FALT) of NPs was measured by monitoring the influence of ionic strength on *ξ*. Different amounts of NaCl stock solutions were added to NPs dispersed in water (250 μg/ mL) and the zeta potential of the samples was measured. A plot of ln *ξ* against 3.33*[NaCl]^0.5^ gives a straight line where the slope represents the thickness of the shell in nm [32].

To evaluate the stability of NPs under physiologically relevant conditions, a known amount of NPs (1 mg) was diluted in PBS at pH 7.4 (NPs concentration 1 mg/mL) and incubated at 37 °C for different times. Size measurements of the samples were taken after 15 min, 1, 2, and 24 h of incubation as described above.

### Dosage of folate exposure

Detection of folate on the NP surface was accomplished by incubating NPs with a specific mAB-antiFol (monoclonal anti-folic acid antibody produced in mouse, Sigma Aldrich) and determining its amount using the Bradford Assay. Briefly, 0.1 mL of NPs (5 mg/mL) were mixed with 0.1 mL of mAB-antiFol solution (34 μg/mL) in 10 mM PBS at pH 7.4, and the resulting solutions incubated for 1 h under mild shaking. The samples were then washed twice with PBS to remove the excess antibody after centrifugation at 270,000 × *g* for 20 min and finally re-dispersed in 0.5 mL of PBS followed by the addition of 500 μL of Bradford reagent. The resultant solutions were heated at 60 °C for 30 min and analyzed by UV spectrophotometry at 595 nm (Shimadzu UV 1800) according to the manufacturer’s instructions. Using a calibration curve of free mAB-antiFol in PBS, the percentage of mAB bound to NP surface was determined. Each assay was repeated 3 times (each with *n* = 3 samples) and the average value was taken as the representative.

### Interaction of NPs with fetal bovine serum

Interaction of NPs with FBS was assessed by fluorescence spectroscopy to determine the “quenching” effect of NPs on the ability of specific residues of the protein to emit light. For the experiment, 200 μL of NPs (1 mg) were mixed with 800 μL of RPMI cell culture medium enriched with 10% FBS, and incubated at RT for 1 h. Then, the emission spectra were acquired (Ex = 278 nm) (RF-6000, Shimadzu Corporation, Japan).

### Generation of spheroids and NP penetration

Multicellular spheroids of KB cells (ATCC, USA) were generated using the liquid overlay method, as previously reported [33]. After 3 days of growth, the spheroids had reached a diameter of about 500 μm and were used for the following experiments.

Three-day-old KB spheroids were treated for 48 h with 50 µg/mL of DiO-loaded NPs. The localization/penetration of NPs was evaluated by confocal microscopy (Leica SP5) by transferring the spheroids from 96-well plates to 35 mm cell imaging dishes and washing them twice with PBS before visualization. Images of about 20 different focal planes (*z*-stack 10 μm) were acquired from the top to the bottom of the spheroid using a 10X objective. DiO fluorescence was revealed using a 488 nm laser as the excitation source and emission filters set from 505 to 550 nm. Maximum projection images were obtained with the software LAS AF Lite by superimposing the images of the 20 acquired focal planes. Furthermore, a 3D reconstruction of the distribution of the fluorescence signal in the equatorial plane of spheroids was obtained using the software ImageJ.

### Cytotoxicity in spheroids

For cytotoxicity, spheroids were incubated for 48 or 72 h with 100 μL of fresh medium containing 10% FBS and increasing concentration of free DTX or DTX-loaded NPs. Cell viability was measured at the end of incubation times using the CellTiter-Glo® 3D Cell Viability Assay (Promega), as previously reported [34]. During the experiment, the spheroid morphological changes were monitored with a bright-field microscope (DMI6000B, Leica) equipped with a DCF365FX camera.

### Zebrafish handling and xenotransplantation

Experiments were performed at the Zebrafish Facility of the University of Padova, under ethical authorization 407/2015-PR. Embryos were obtained from natural spawning of *Casper* mutants (a9-w2 double mutant line; ZFIN IDs: ZDB-ALT-980203–444, ZDB-ALT-990423–22) and *fli1a:EGFP* transgenic adults (ZFIN ID: ZDB-ALT-011017–8), raised according to standard protocols [35] and staged according to Kimmel et al. [36] For xenotransplantation, embryos were mechanically dechorionated at 2 days post-fertilization (dpf), anesthetized with 0.16 mg/mL tricaine, and placed along the lanes of a microinjection mold, immersed in 2% methylcellulose/fish-water. KB cells were suspended at a density of 1 × 10^6^/mL and stained with 5 µg/mL of DiI Vybrant Cell-Labeling Solution (Molecular Probes) for 20 min at 37 °C. Stained cells were loaded into a glass capillary needle and microinjected into the yolk (about 100 cells/embryo), using a WPI PicoPump apparatus. Twenty-four hours after tumor transplantation (3 dpf), embryos were microinjected using the same procedures described above with an aqueous solution of phenol red (control), free DTX, DTX-loaded NPs (DTX-DBL, DBL_Fol_, DBL_Fol/aFLT1_), and unloaded DBL_Fol/aFLT1_. Each embryo was injected with a DTX dose of about 2.5 ng; each experimental group of treatment was constituted of 25 embryos. Xenotransplanted embryos were grown at 33 °C and monitored daily starting from the injection day up to 1 week post-injection (experimental endpoint, 9 dpf). Non-xenografted embryos injected with DTX formulations were observed in parallel. Analyses included mortality rate, in situ (yolk) or metastatic (extra yolk) cancer cell location, and tumor size reduction evaluation, performed by cell counting/signal quantification using ImageJ software. Imaging was performed using a Leica MZFLIII fluorescence-dissecting microscope equipped with a Leica DFC7000T camera.

For the analysis of neo-angiogenesis, the *fli1a:EGFP* transgenic line, having blood vessels and micro-vessels visible in green fluorescence, was used. The embryos (2 dpf) were injected with KB cells and 1 h later with DTX or DTX-DBL_Fol/aFLT1_. Tumor and blood vessels were analyzed 48 h later by fluorescence microscopy.

### Statistical analysis

The Primer software for biostatistics (McGraw-Hill, Columbus, USA) was used for statistical analysis of the data. The data are expressed as means ± standard deviations (SD) for at least 3 independent experiments. The difference between groups was evaluated with the Student’s *t*-test and was considered significant for *p* < 0.05.

## Results and discussion

### NP preparation and characterization

**Fig. 1:**
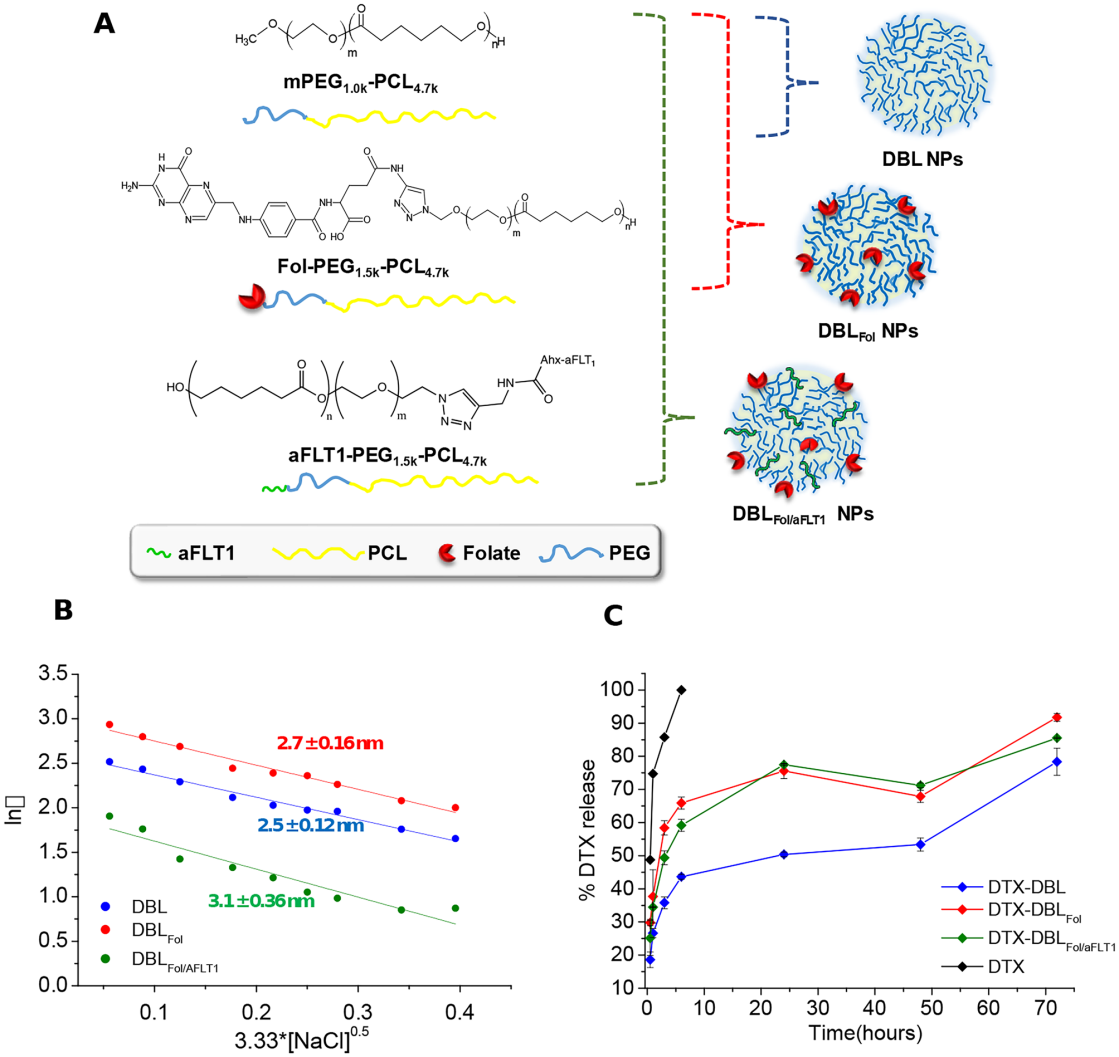
**A** Chemical structure of the copolymers employed to prepare NPs. **B** Representative Fixed aqueous layer thickness (FALT) measurements evaluated by monitoring the zeta potential of NPs dispersed in NaCl solutions in water ([NPs] = 250 μg/mL): the slope of the straight line represents the thickness of the outer hydrophilic shell expressed in nm. **C** Docetaxel (DTX) release from NPs in PBS 10 mM pH 7.4, at 37 °C evaluated by the dialysis method. Results are the mean values ± SD of three measurements carried out on three different NPs batches

In this work, we conceived a multifunctional NP for cancer therapy applications, in which the active targeting of the therapeutic cargo is complemented with anti-angiogenic effects. Accordingly, we designed a NP delivering DTX based on a mix of PCL-PEG copolymers, some modified at the PEG end chain with either aFLT1 or Fol to obtain the multifunctional nanoplatform with double decoration (DBL_Fol/aFLT1_) (Fig. 1A). For comparison purposes, unmodified NPs of PEG-PCL (DBL) or Fol-PEG-PCL/PEG-PCL (DBL_Fol_) were prepared. Unloaded or DTX-loaded NPs were obtained by the solvent diffusion/evaporation method. The ratio between Fol-PEG-PCL/PEG-PCL and Fol-PEG-PCL/aFLT1-PEG-PCL was selected based on our previous findings [17, 30]. It is worth noting that the manipulation space of the formulation was limited by aggregation in the nanoprecipitation step when Fol-PEG-PCL or aFLT1-PEG-PCL amount was increased and that of PEG-PCL was decreased. The overall properties of the NPs are reported in Table 1.

**Table 1:** Properties of unloaded and DTX-loaded NPs

Formulation	*D*_H_ (nm ± SD)	PI	ζ (mV ± SD)	Yield (% ± SD)	DTX actual loading (mg DTX/100-mg NPs)	DTX entrapment eff.^a^ (%)
DBL	78 ± 2	0.1	−12 ± 0.1	68 ± 4	–	–
DBL_Fol_	84 ± 5	0.1	−18 ± 6	52 ± 3	–	–
DBL_Fol/aFLT1_	101 ± 8	0.2	10 ± 3	54 ± 1	–	–
DTX-DBL	92 ± 5	0.2	−10 ± 1	72 ± 6	8.8 ± 0.9	98 ± 4
DTX-DBL_Fol_	84 ± 8	0.1	−19 ± 4	61 ± 4	8.7 ± 1.5	98 ± 10
DTX-DBL_Fol/aFLT1_	101 ± 5	0.1	−9 ± 2	55 ± 3	8.5 ± 0.6	96 ± 6

^a^Theoretical loading of DTX was 9 mg DTX per 100 mg of NPs

All the formulations show a *D*_H_ between 78 and 101 nm, a low polydispersity index, and a negative ζ. NPs prepared from copolymer mix (DBL_Fol_ and DBL_Fol/aFLT1_) are larger than DBL, suggesting that segregation of hydrophilic blocks in the PCL core occurs [17].

In Fig. 1B, the values of *ξ* in NaCl solutions at different concentrations are shown. The thickness of the external hydrophilic PEG shell of NPs, which is the slope of the regression lines in absolute value, is similar for the NPs tested, thus suggesting that the presence of surface motifs has a slight impact on the conformation of the PEG present on the surface of NPs (Fig. 1B). DTX entrapment in the lipophilic core of NPs (9% theoretical loading) is almost complete and does not affect the colloidal properties of NPs.

The release of DTX from NPs (Fig. 1C), evaluated in 10 mM PBS at pH 7.4 and 37 °C by dialysis, is bimodal for all the formulations. The burst in the 0–6 h interval is higher for NPs obtained from copolymer mix than bare DBL, in line with the hypothesis that some hydrophilic blocks in the PCL core matrix decrease the crystallinity of NPs and increase the diffusivity of the entrapped DTX. Release of DTX is completed in ca. 72 h for all the formulations tested. Free DTX is quantitatively released in the external medium in ca. 6 h, ensuring sink conditions.

**Fig. 2:**
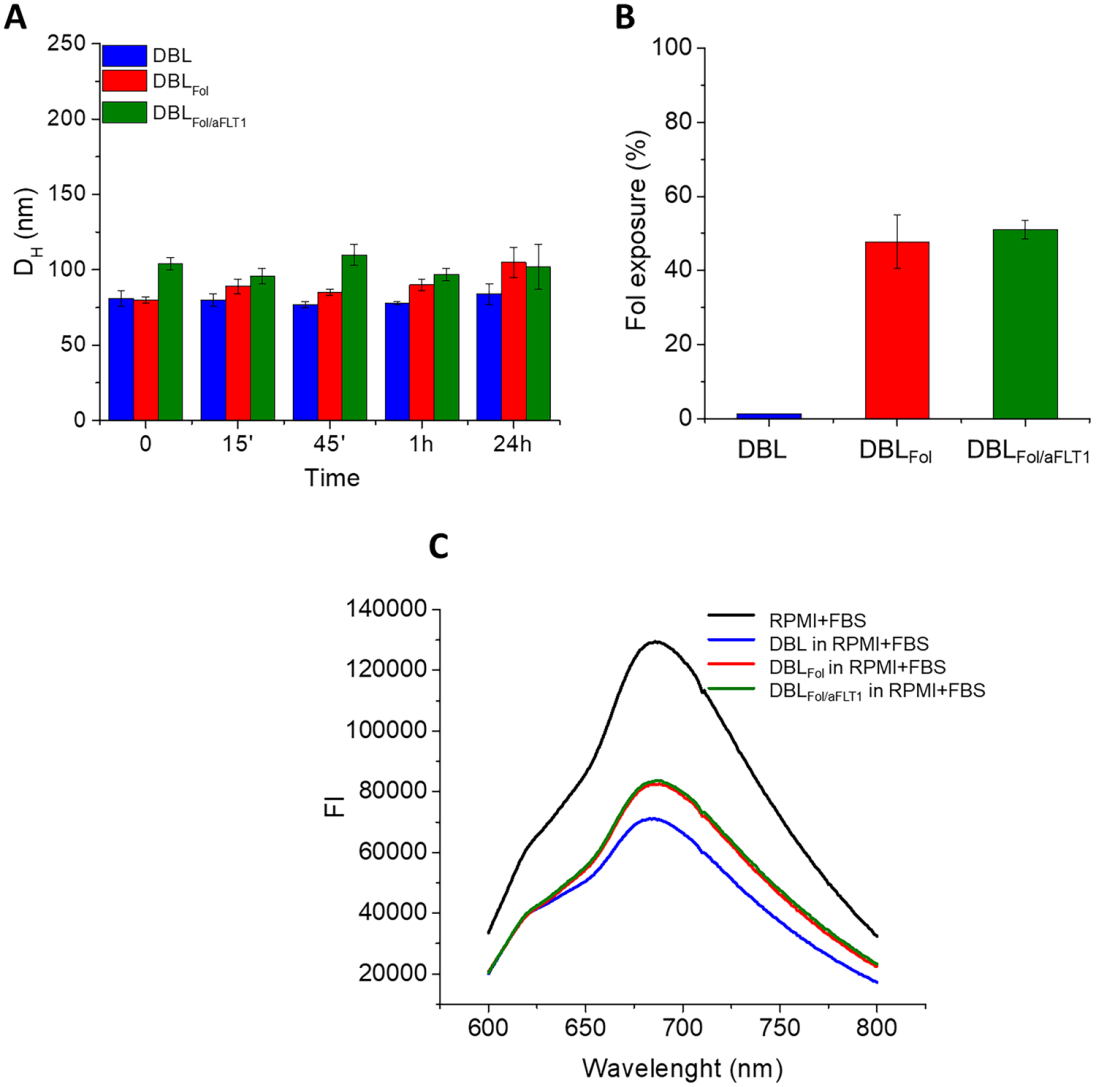
**A** Hydrodynamic diameter of NPs after incubation in PBS pH 7.4 at 37 °C at different time points ([NPs] = 1 mg/mL). **B** Percentage of folate exposure on NP surface measured by incubating NPs (5 mg/mL) with a monoclonal anti-folic acid antibody. Results are the mean values ± SD of three measurements carried out on three different NPs batches. **C** Fluorescence emission spectra of RPMI with 10% of FBS at Ex = 278 nm in the presence of DBL NPs, DBL_Fol_ NPs and DBL_Fol/aFLT1_ ([NPs] = 1 mg/mL). The reduction of the emission of RPMI + FBS indicates an interaction between NPs and the protein

As shown in Fig. 2A, NPs show excellent stability in PBS pH 7.4 at 37 °C, employed as simulated biological studies until 24 h of incubation. Then, the folate exposure on the NP surface was checked through the binding with a specific mAB against folate. As evident in Fig. 2B, a 40% antibody was able to bind DBL_Fol_ and DBL_Fol/aFLT1_ NPs, independently by the presence of the anti-angiogenic peptide. The experiment was carried out also on untargeted NPs as a negative control.

Then, before moving to in vitro and in vivo experimentation, we checked NPs interaction with proteins present in simulated biological fluids. Thus, NPs were dispersed in RPMI cell culture medium added with 10% fetal bovine serum (FBS) and the quenching of FBS emission after excitation at 278 nm was evaluated. We found a similar interaction between the proteins and the different formulations, slightly pronounced in the case of DBL_Fol/aFLT1_ NPs and DBL_Fol_ (Fig. 2C). Finally, we confirmed the elevated anti-angiogenic activity of aFLT1 displayed on the surface of DBL_Fol/aFLT1_: compared with DBL and DBL_Fol,_ which, as expected, did not show any capacity to inhibit endothelial tube formation (Fig. S1), DBL_Fol/aFLT1_ significantly affect all tube parameters (e.g. the number of junctions, master segments and meshes).

### In vitro behavior of NPs in KB spheroids

**Fig. 3:**
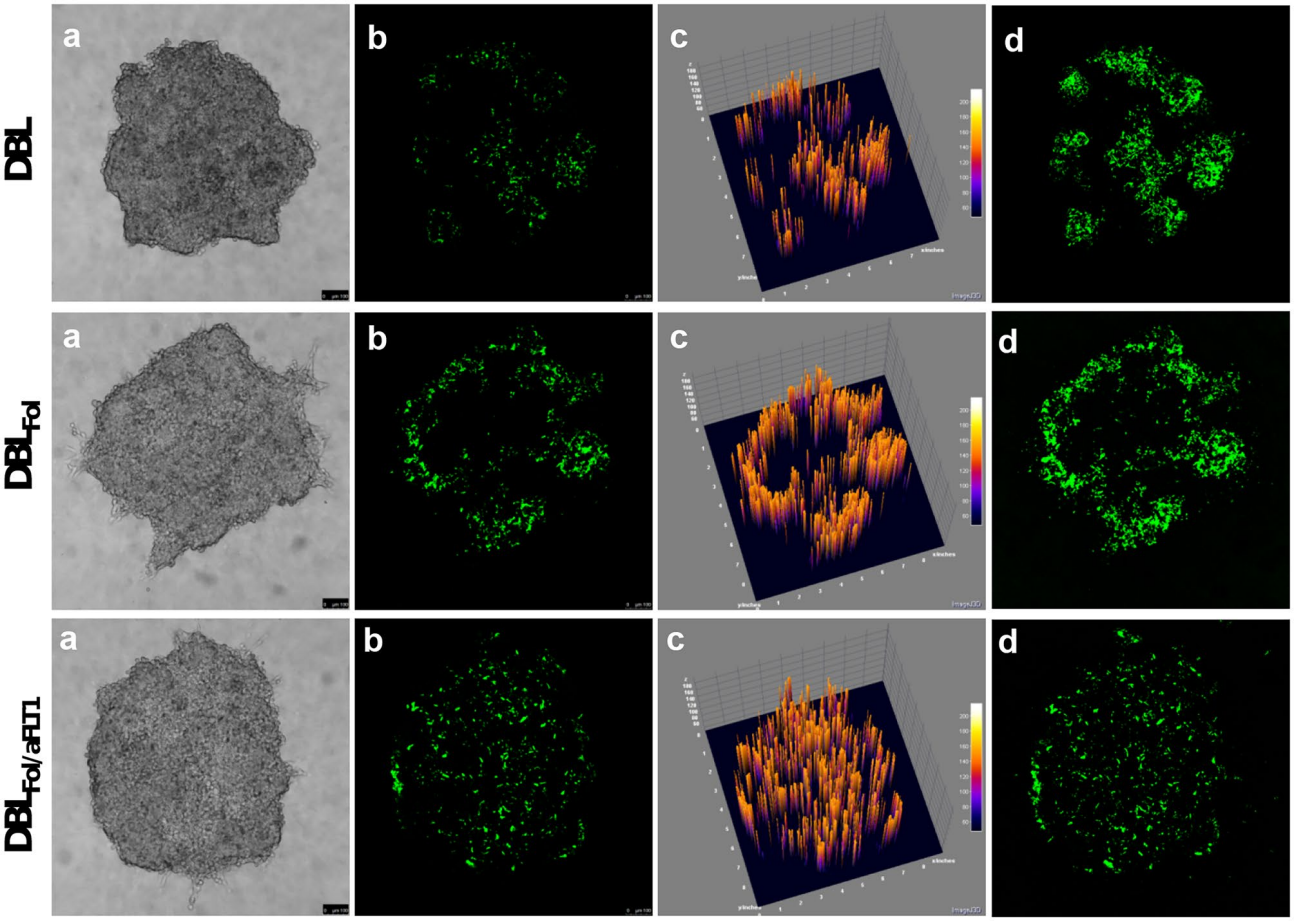
Confocal images of KB spheroids after treatment with DiO-loaded NPs (50 µg/mL) for 48 h: **a** bright-field; **b** DiO fluorescence acquired in the equatorial plane of the spheroid; **c** 3D reconstruction of the distribution of the fluorescence signal in the equatorial plane of the spheroid; **d** maximum projection obtained by superimposing the images of the 20 acquired focal planes. Scale bars: 100 μm

**Fig. 4:**
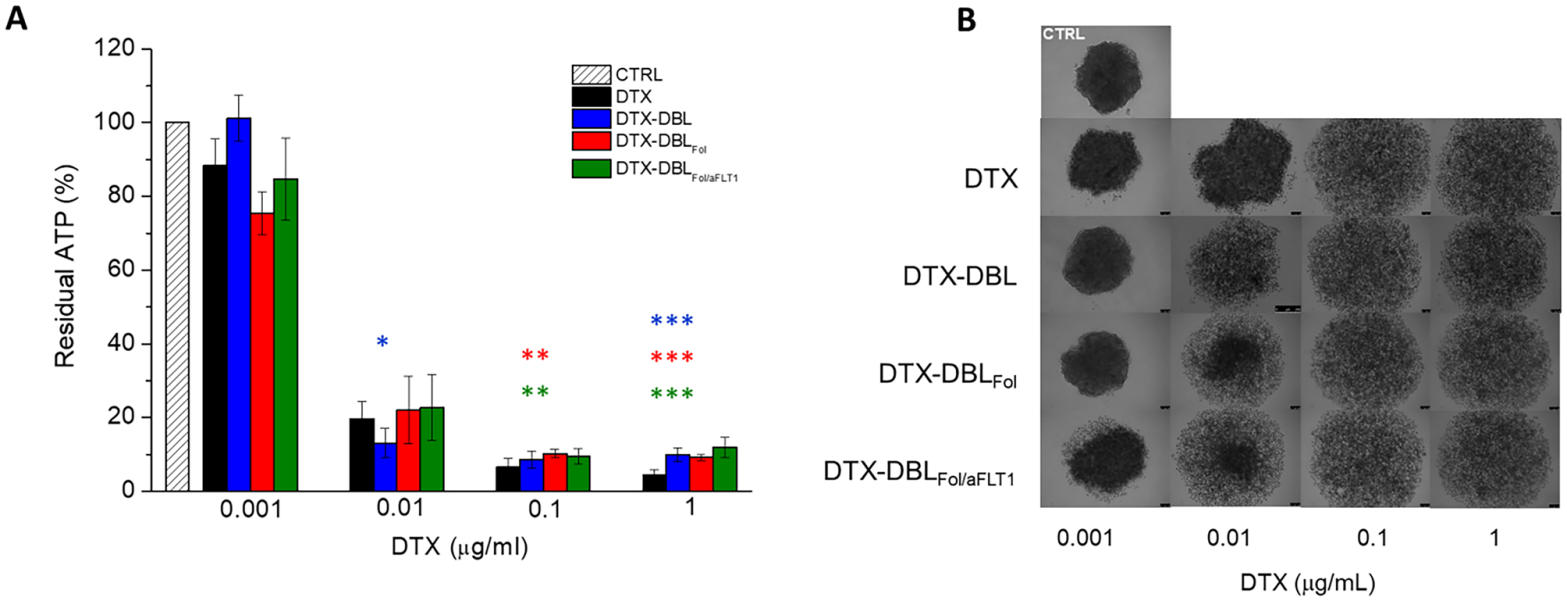
Cytotoxicity of DTX-loaded NPs in KB spheroids. **A** Percentage of residual ATP in the spheroid measured using the CellTiter-Glo® 3D Cell Viability Assay after 72 h of treatment with DTX-loaded NPs or with the free drug ([DTX] = 0.001–1 μg/mL). Data are mean values ± SD of at least three independent experiments carried out in triplicate. *p < 0.05, **p < 0.01, ***p < 0.001 vs. free DTX (Student’s *t*-test). **B** Bright-field images of spheroids acquired by confocal microscopy after 72 h of treatment with DTX-loaded NPs at different doses. Scale bars: 100 μm

The behavior of NPs was assessed in FRα positive KB cells cultured as avascular spheroids to mimic more closely the in vivo three-dimensional tumor architecture and the enrichment in extracellular matrix components, which may affect drug distribution and efficacy [37]. The penetration of DBL_Fol_ and DBL_Fol/aFLT1_ NPs loaded with DiO (properties in Table S1) in KB spheroids is shown in Fig. 3. Confocal images, taken at the median plane of the spheroids after 48 h of incubation, evidence a different distribution pattern depending on NP decoration. Due to the high lipophilicity of DiO, we assume that the green fluorescence visible in the cell aggregates can be identified with NPs. While fluorescence is randomly distributed for DBL, the green signal is confined in the outer rims for DBL_Fol_ and uniformly distributed in the entire spheroid for DBL_Fol/aFLT1_. Tridimensional plots showing fluorescence signal intensity (Fig. 4, panels c) and maximum projections (Fig. 4, panels d) revealed that NPs accumulated in the order DBL < DBL_Fol/aFLT1_ < DBL_Fol_ as compared to the total fluorescence. The prevalent location of DBL_Fol_ in the spheroid rim is in line with the so-called “barrier effect” observed for NPs targeting cancer cells receptors. Surprisingly, the parallel presence of aFLT1 on NP surface attenuates this effect and promotes DBL_Fol/aFLT1_ penetration in the whole-cell aggregate, likely due to a shielding effect exerted by peptide chains.

A concentration- and time-dependent cell viability reduction was measured upon treatment of 2D cultures of both KB and HUVEC with DTX and DTX-loaded NPs (Fig. S2), after demonstration of the tolerability of unloaded NPs (Fig. S3). Cell viability of DTX-DBL_Fol_ and DTX-DBL_Fol/aFLT1_ vs. DTX-DBL was not modified by surface decoration, probably due to the weak contribution of FR-mediated NPs endocytosis as compared to non-specific endocytosis, as we previously reported for monolayer cell cultures [17].

The cytotoxicity of DTX-loaded NPs measured by the 3D Glo Assay in KB spheroids after 72 h of treatment is reported in Fig. 4. Results are expressed as residual ATP content *vs.* untreated cells as a significant parameter to measure viability reduction and toxicity. The incubation of KB spheroids with DTX-loaded NPs determines a sharp cell viability reduction, although no significant differences between the formulations were observed independently of the time (see also Fig. S4 reporting cell viability of spheroid after 48 h of incubation). The cytotoxicity of free DTX is dose- and time-dependent and confirmed by the bright-field images of the treated spheroids (Fig. 4B and Fig. S4B), highlighting that the disaggregation of the spheroids accompanies the progressive loss of viability. As expected, the extent of cell release and mortality started from the periphery to the core of the spheroids, especially with DTX-DBL_Fol_ and DTX-DBL_Fol/aFLT1_ at a drug concentration of 0.01 μg/mL at both 48 and 72 h. Unloaded NPs were not toxic to cell spheroids (Fig. S5).

The different pattern of cellular aggregate dissociation is consistent with the observation that Fol decoration of NPs promoted the accumulation mainly at the periphery of the spheroid and exerted cytotoxic effects prevalently in the rim. The fact that cytotoxicity of NPs is comparable with that of DTX demonstrates once again that a complex interplay between DTX release and NP transport/accumulation in the cancer cell aggregates occur, making it difficult to draw conclusions on the superior activity of NPs.

### In vivo anti-tumor and anti-angiogenic activity of NPs in zebrafish embryos

As the mammalian models, zebrafish has striking evolutionary conservation of disease-related genes and pathways in humans, and thus both models are widely employed in cancer research and nanomedicine screening. The choice of using *Danio rerio* embryos as an exploratory in vivo non-mammalian model to study tumor regression and angiogenesis after treatment with NPs was encouraged by several preliminary considerations [38],: (i) the transparency of the embryo, which allows easy observation of tumor development, angiogenesis, and metastasis in real-time; (ii) high physiologic and genetic similarity to mammals [38]; (iii) low cost of maintenance and ease of manipulation as compared to rodents. Furthermore, the immune system of embryos is immature up to 11 dpf, thus avoiding or delaying the rejection of xenotransplants [39]. The availability of transgenic models over-expressing fluorescent proteins (e.g., vascular proteins) also offers a tool to monitor neo-angiogenesis easily [40, 41]. Recently, zebrafish has emerged as a useful preclinical cancer model for the preliminary screening of nanomedicines [42], mainly because it allows the rapid and high-throughput in vivo evaluation of nanoformulations with more sustainable costs than rodents and satisfying at the same time the 3Rs legislative guidelines. Indeed, conventional 2D and 3D in vitro cancer models are often associated with investigations using the xenografted zebrafish model to assess NP interactions in vivo under more complex and representative biological conditions. Although no final correlation between zebrafish and mice cancer models has been drawn, the potential of zebrafish cancer avatars and how these models compare with and complement mouse xenografts and human organoids has been recently reviewed [43].

**Fig. 5:**
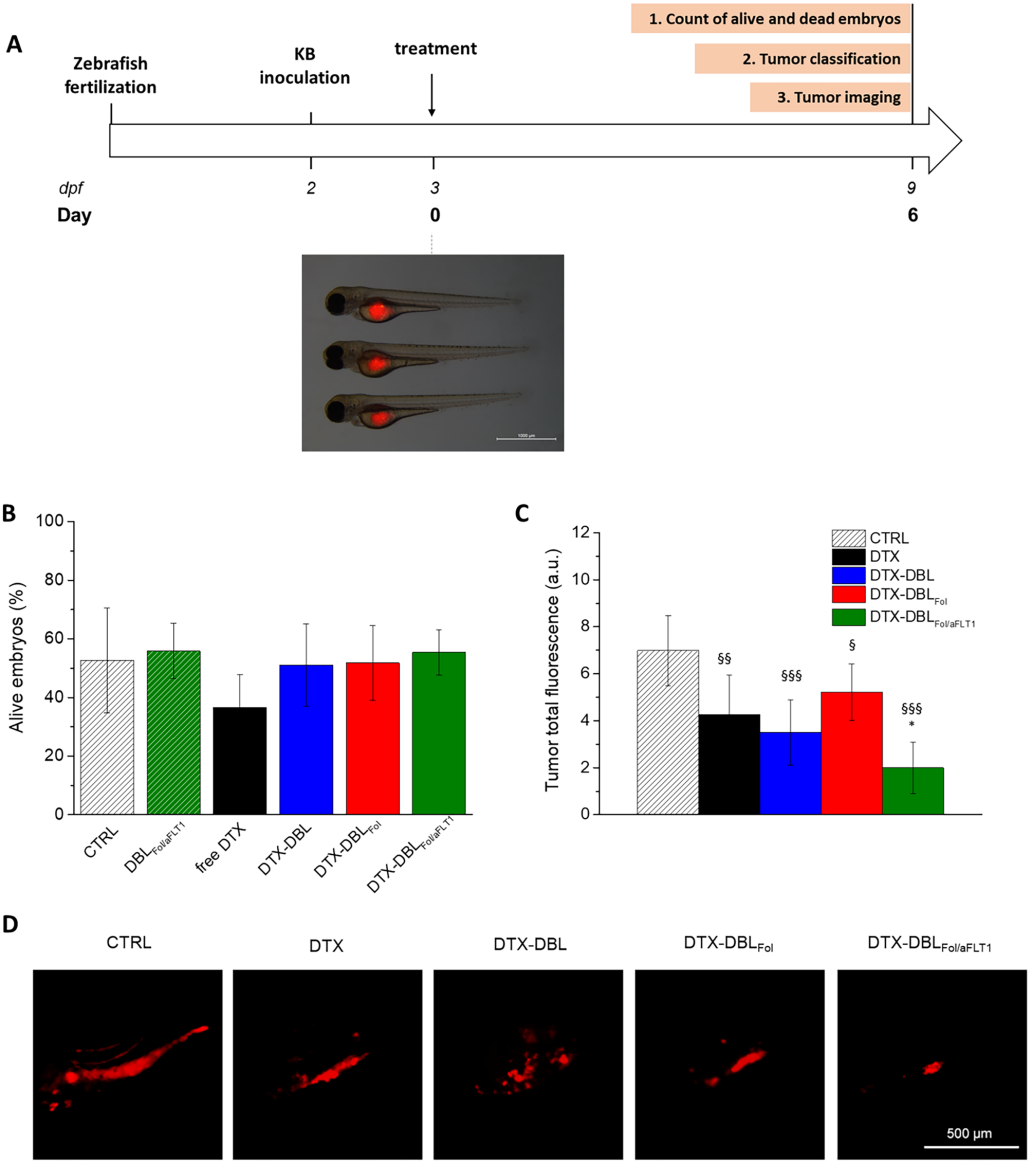
Toxicity and activity of DTX-loaded NPs in the zebrafish embryo xenograft model. **A** Timeline of the in vivo experiments. The microscopy image shows 3 embryos xenografted with KB cells (red fluorescence). **B** Toxicity of NPs in xenograft zebrafish embryos injected with DTX and DTX-loaded NPs ([DTX] = 2.5 ng/animal) after 6 days from the treatment. **C** Tumor volume reduction after 6 days from the treatment with DTX-loaded NPs ([DTX] = 2.5 ng/animal). Total tumor fluorescence quantified from images. ^§^*p* < 0.05; ^§§^*p* < 0.01; ^§§§^*p* < 0.001 vs. ctrl; *p < 0.001 vs. free DTX. **D** Representative images of KB cells xenotransplanted in the embryo yolks. Scale bars: 500 μm

The experimental procedure in zebrafish adopted here is based on the injection of both tumor cells and NPs in the yolk of the embryo by microinjection to achieve local effects as recently demonstrated for glioblastoma [44]. The set-up of the experiments in albino zebrafish embryos xenografted with KB cells is outlined in Fig. 5A. The yolk region of 2-dpf embryos was inoculated with DiI-labeled KB cells, and the following day re-injected with DTX, DTX-loaded NPs, and unloaded DBL as control. At the time of NP inoculation, KB cells are confined in the yolk of the embryos forming a single mass with spherical shape and a mean diameter of about 600–800 μm, a size close to that of spheroids tested in vitro (Fig. 5A).

Animal survival analysis revealed that, in the case of embryos xenografted with KB cells, the percentages of alive animals decreased to 50% for the control group and all the variants of DTX-loaded NPs (Fig. 5C) as compared with 70% for the same groups in non-xenografted animals (Fig. S6). Remarkably, DTX treatment reduced the percentage of alive embryos from 70 to 40% in the non-xenograft embryos (Fig. S6), thus highlighting the role of NPs in protecting the embryo from the high systemic toxicity of DTX.

The efficacy of DTX-loaded NPs in reducing effectively tumor masses was assessed on selected animals bearing a single mass in the yolk 6 days post-treatment. As clearly visible in Fig. 5C, a high reduction of KB-associated fluorescence in all the treated groups compared to control was observed. Notably, the greatest extent of tumor reduction was obtained with DTX-DBL_Fol/aFLT1_, which means that an improvement of DTX efficacy occurs due to both the targeting and the anti-angiogenic motifs. Noteworthy, the presence of Fol on NPs surface did not result in any tumor size reduction improvement compared to untargeted NPs. In fact, three different tumor conditions (indicated as tumor phenotypes) occurred after 6 days of treatment as exemplified with representative images in Fig. S7A: single masses located in the yolk (a), multiple masses located in the yolk (b), tumor masses in the yolk plus distal masses (metastasis-like masses) outside yolk region (c). Cell migration from the yolk region is considered indicative of cell invasiveness [45]. The percentages of tumor phenotypes are reported in Fig. S7B. Overall, the extent of cell invasiveness was relatively low (~ 20%), and only DTX-DBL_Fol_ significantly reduced KB cell spread. Moreover, while the number of animals with single or multiple masses in the yolk was comparable in the control group, the treatment with free DTX and DTX-DBL_Fol/aFLT1_ completely abolished the formation of multiple tumor masses, with about 70% of alive embryos having a single tumor mass. Unlike in vitro observations, tumor regression observed in zebrafish highlighted the superior activity of DTX-DBLFol/aFLT1 compared to free DTX accompanied by a concomitant decrease of DTX systemic toxicity.

**Fig. 6:**
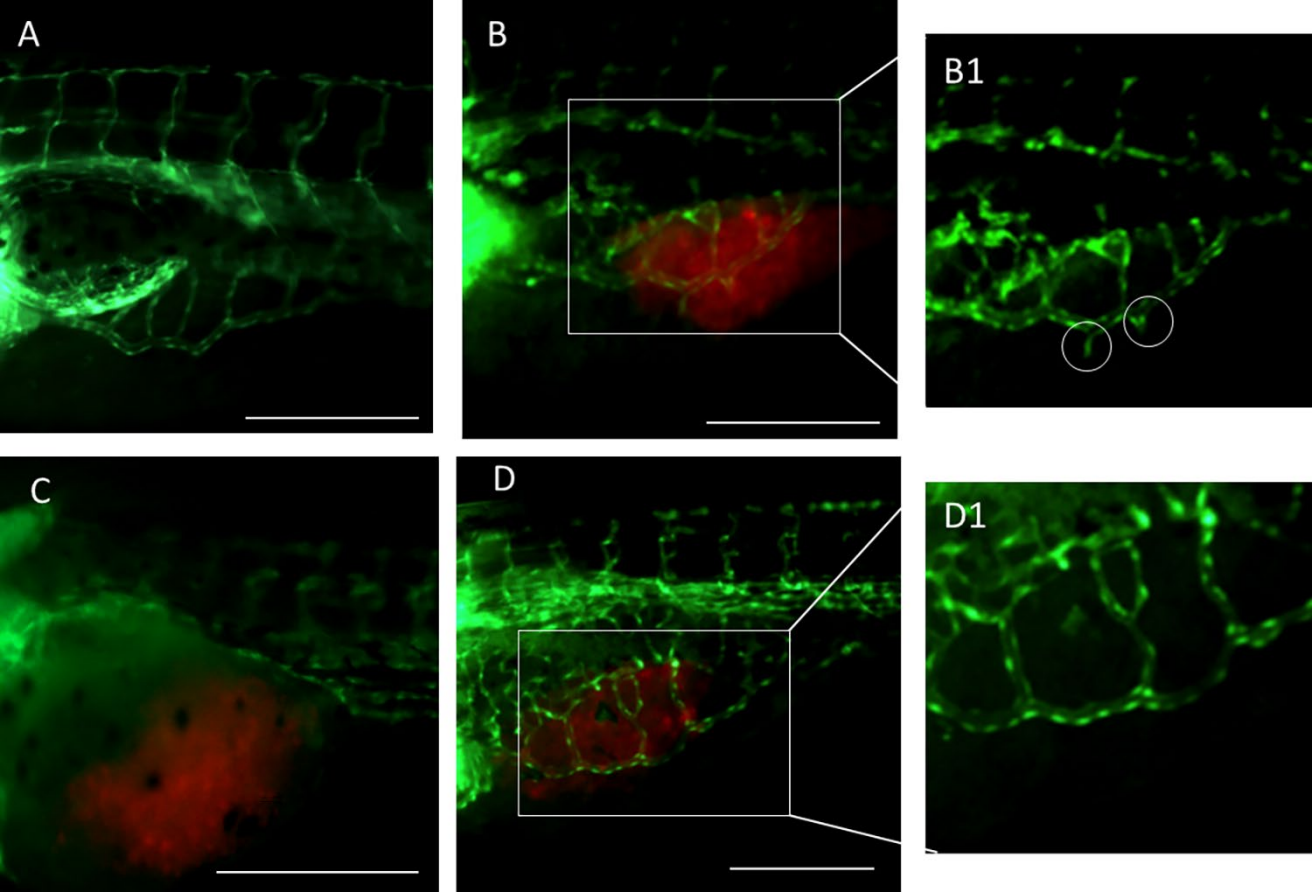
Vasculature analysis of *fli1a:EGFP* embryos after two days of treatment with nanoparticles (NPs) ([DTX] = 2.5 ng/animal). Fluorescence microscopy images of untreated embryos without (**A**) or with KB xenografted cells (**B**); embryos with KB xenografted cells and treated with free DTX (**C**) or DTX-DBL_Fol/aFLT1_ (**D**). B1 is a magnification showing the presence of vascular branches (white circles) sprouting toward tumor masses in untreated embryos. In the magnification, D1 vascular branches are not visible due to the anti-angiogenic effect exerted by DTX-DBL_Fol/aFLT1_. Scale bar: 250 μm. Red: tumor cells stained with DiI; green: zebrafish embryo vasculature

To understand if this effect was related to the anti-angiogenic activity of DTX-DBL_Fol/aFLT1_, we employed a *fli1a:EGFP* transgenic embryo model. We analyzed eventual macroscopic alterations of blood vessel features 48 h after NPs injection and compared the results to those collected from untreated or DTX-treated embryos. Figure 6A, B are representative images of an embryo (untreated) xenografted or not with KB cells, respectively. In both embryos, the intact sub-intestinal vessels (SIVs), with a shape like a basket projected inside the yolk region, are completely formed at this development stage. As clearly visible in Fig. 6, panel B1, and according to some recent pieces of evidence [46], the injection of tumor cells induces pro-angiogenic effects by promoting the formation of additional branches (white circles), which essentially sprout toward the tumor mass.

Interestingly, the treatment of the xenografted embryos with DTX-DBL_Fol/aFLT1_ prevented the formation of these branches (Fig. 6D, D1), thus suggesting an active role of aFLT1. After DTX treatment, the classic basket structure was not formed (Fig. 6C). The vessels were utterly disorganized within the swollen yolk sack, in line with its superior systemic toxicity.

Taken together, our results demonstrate that tumor regression due to DTX-DBL_Fol/aFLT1_ is due to DTX cytotoxicity combined with a significant anti-angiogenic capacity of NPs, which normalize vessels rather than disrupt entirely endothelial cells. Notably, while we were unable to measure any active role of the folate moiety in the in vitro model, DTX-DBL_Fol_ NPs attenuated the overall toxicity of DTX, demonstrating both an increased anticancer effect and a decreased spread of metastasis in the xenografted zebrafish. The latter observation stresses the importance of the in vivo studies to test NPs efficacy, even using experimental models alternative to rodents as zebrafish, and highlights that three-dimensional in vitro models as spheroids are by themselves only poorly predictive of in vivo behavior. In perspective, our results can be considered significant for ovarian cancer, which is eligible for a local treatment aimed to target avascular spheroids dissemination in the peritoneum and to block angiogenesis involved in distal metastasization.

## Conclusion

We have herein reported the development of PEGylated NPs surface-modified with folate/aFLT1 motifs as a strategy to potentiate the activity profile of DTX and to limit its systemic toxicity. The presence of folate on the NP surface did not alter aFLT1 anti-angiogenic activity but affected their penetration in KB spheroids. No significant impact of NP composition on cytotoxicity in the spheroids was found. Remarkably, DTX activity was enhanced in a xenografted zebrafish embryo model when delivered through NPs bearing folate/aFLT1 motifs, whereas DTX toxicity was decreased. To our knowledge, this is the first demonstration that such double decoration of biodegradable PEGylated NPs is feasible and beneficial to increase the efficacy of the bare PEGylated counterpart. Our findings can pave the way to extend this NP concept to other anti-angiogenic peptides and chemotherapeutics, widening the arsenal of nanoplatforms to fight solid tumors.

## Supplementary Information

Below is the link to the electronic supplementary material.

Supplementary file1 (DOCX 1734 KB)

## Data Availability

The raw data are available from the authors.
