# Supplementary material for: Biodegradable nanoparticles combining cancer cell targeting and anti-angiogenic activity for synergistic chemotherapy in epithelial cancer
Source: Drug Deliv Transl Res. 2022 Jan 1;12(10):2488–500. doi: 10.1007/s13346-021-01090-6 (PMC9458690; doi:10.1007/s13346-021-01090-6)
Supplement: Supplementary file 1 — Supplementary file1 (DOCX 1734 KB) [file 13346_2021_1090_MOESM1_ESM.docx]

**Supplementary material**

**Biodegradable nanoparticles combining cancer cell targeting and anti-angiogenic activity for synergistic chemotherapy in epithelial cancer^†^**

Francesca Moret,^a,#^ Claudia Conte,^b,#^ Diletta Esposito,^b^ Giovanni Dal Poggetto,^c^ Concetta Avitabile,^d^ Francesca Ungaro, ^b^ Natascia Tiso,^a^ Alessandra Romanelli,^e^ Paola Laurienzo,^c^ Elena Reddi ^a,*^ and Fabiana Quaglia^b,c*^

^a^ Department of Biology, University of Padova, 35121 Padova, Italy

^b^ Department of Pharmacy, University of Napoli Federico II, 80131 Napoli, Italy

^c^ Institute for Polymers, Composites and Biomaterials, CNR, 80078 Pozzuoli, Italy

^d^ Institute of Biostructure and Bioimaging, CNR, 80134 Napoli, Italy

^e^ Department of Pharmaceutical Sciences, University of Milan, 20133 Milano, Italy

^#^ equally contributed

* Corresponding authors

quaglia@unina.it (F. Quaglia), elena.reddi@unipd.it (E. Reddi)

**Table S1.** Properties of DiO-loaded NPs

| **Formulation** | **Size**  **(nm ± SD)** | **PI** | **ζ**  **(mV ± SD)** | **Yield**  **(%± SD)** | **DiO Actual loading**  **(mg DiO/100 mg NPs)** | **DiO**  **Entrapment Eff.**  **(%)** |
| --- | --- | --- | --- | --- | --- | --- |
| DiO-DBL | 95±7 | 0.1 | -11±3 | 75±3 | 0.98±0.02 | 98 ± 3 |
| DiO-DBL_Fol_ | 90±5 | 0.1 | -12±4 | 69±7 | 0.98±0.05 | 98 ± 4 |
| DiO-DBL_Fol/aFLT1_ | 98±6 | 0.1 | -10±3 | 65±6 | 0.97±0.03 | 97 ± 5 |

## S1. *In vitro* endothelial tube formation assay

Geltrex® Matrix (Geltrex^®^ LDEV-Free Reduced Growth Factor Basement Membrane Matrix, Life technologies) was thawed at 4 ^°^C overnight. Each well of pre-chilled 24-well/plates was coated with 100 μl of matrix, incubated, and solidified at 37 °C for at least 30 min. Meanwhile, HUVEC cells (cultured for maximum 4 passages from thawing) were harvested from flasks and seeded (4.8 x 10^4^ cells/well) in M200 supplemented with LVES (Large Vessel Endothelial Supplement, Gibco) for 14 h. During the seed, 50 μg/mL of NPs were added in each well. Wells containing exclusively HUVEC cells were used as positive controls of tube forming cells. At the end of the incubation time, cells were washed with PBS with Ca^2+^ and Mg^2+^, incubated at 37 ^°^C with Calcein AM 2 μM (Molecular Probes) for 15 min, washed again and maintained in M200 medium for all image acquisition period. Tube formation was analyzed using an inverted fluorescence microscope (DMI4000, Leica) at 5X or 10X magnification. Image analysis was performed from randomly selected fields using a dedicated plug-in (Angiogenesis Analyzer) for Image J, and the percentage of inhibition in treated cells vs. control cells was reported for the formed numbers of junctions, master segments, and meshes.

**
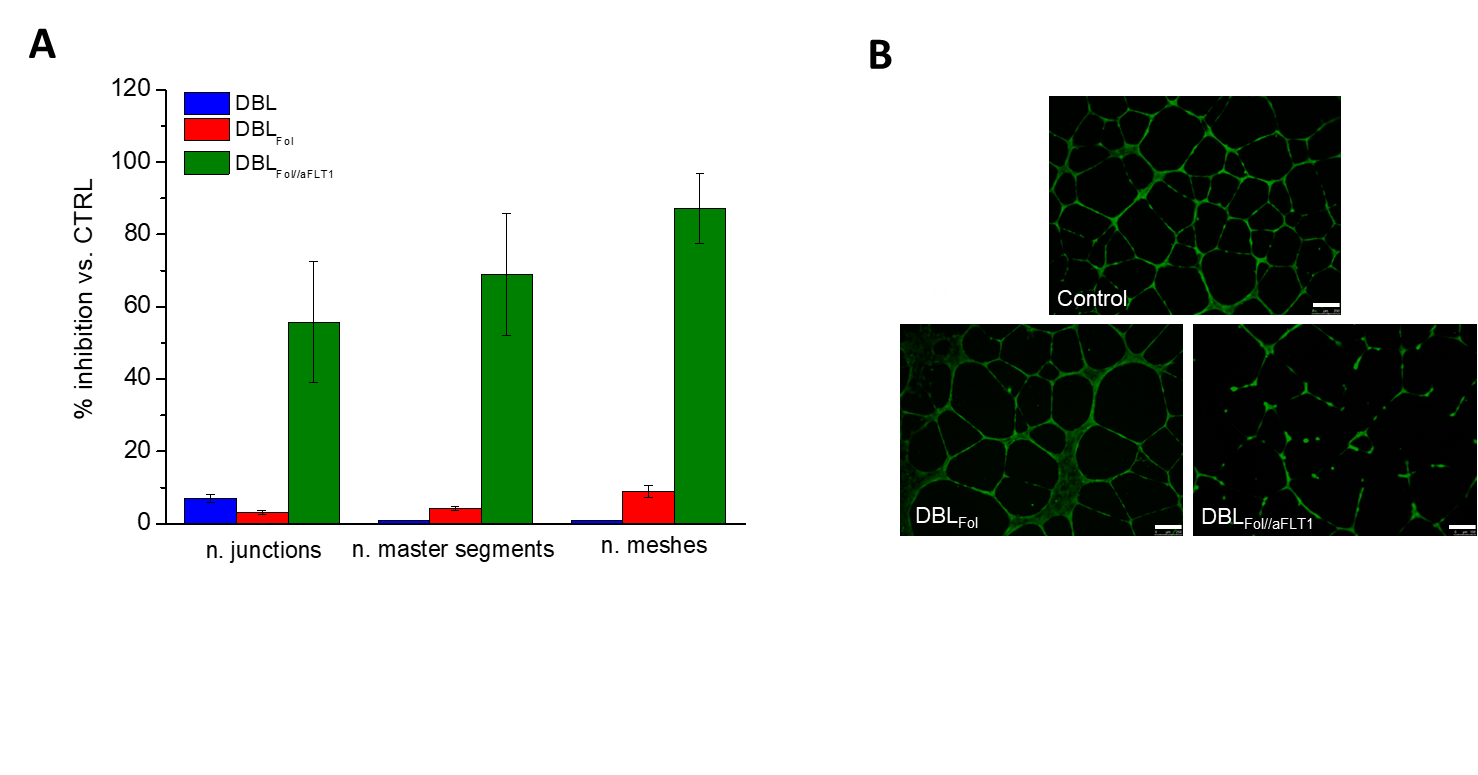
**

**Figure S1.** *In vitro* endothelial tube formation assay. Inhibition of tube formation in HUVEC cells after incubation with 50 μg mL^-1^ of unloaded nanoparticles (NPs) for 18 h and B) representative fluorescence images of tubes. Scale bars: 250 μm.

## S2. Cytotoxicity on monolayer cell cultures

Cell lines were obtained from American Type Culture Collection (ATCC, USA). HUVEC were cultured in Medium-200 supplemented with antibiotics (100 U/mL Penicillin G and 100 µg/mL Streptomycin) and Low Serum Supplement Kit (Life Technologies). KB cells were grown in MEM supplemented with 10% FBS, while during the experiments with NPs were seeded in Fol-deficient RPMI. The viability of cells treated with increasing concentrations of DTX-loaded NPs was measured with the MTS assay (CellTiter 96^®^ AQueous One Solution Cell Proliferation Assay, Promega Co., USA). Free DTX dissolved in DMSO was used as control. Cell viability was also measured after treatment with unloaded NPs. Cells (5 × 10^3^ KB or 7 × 10^3^ HUVEC) were seeded in 96-well plates (24 h of growth) and incubated with the various formulations dispersed in medium with 10% FBS. After 48 and 72 h of incubation, the medium was replaced with 100 μL of serum-free medium and 20 μL of the CellTiter 96^®^ reagent and the wells incubated for 1 h at 37 ^°^C. The absorbance at 492 nm was measured with a Multiskan Go (Thermo Fischer Scientific, USA) plate reader, and the viability of treated cells was expressed as the percentage of the absorbance of control cells considered as 100% viability.


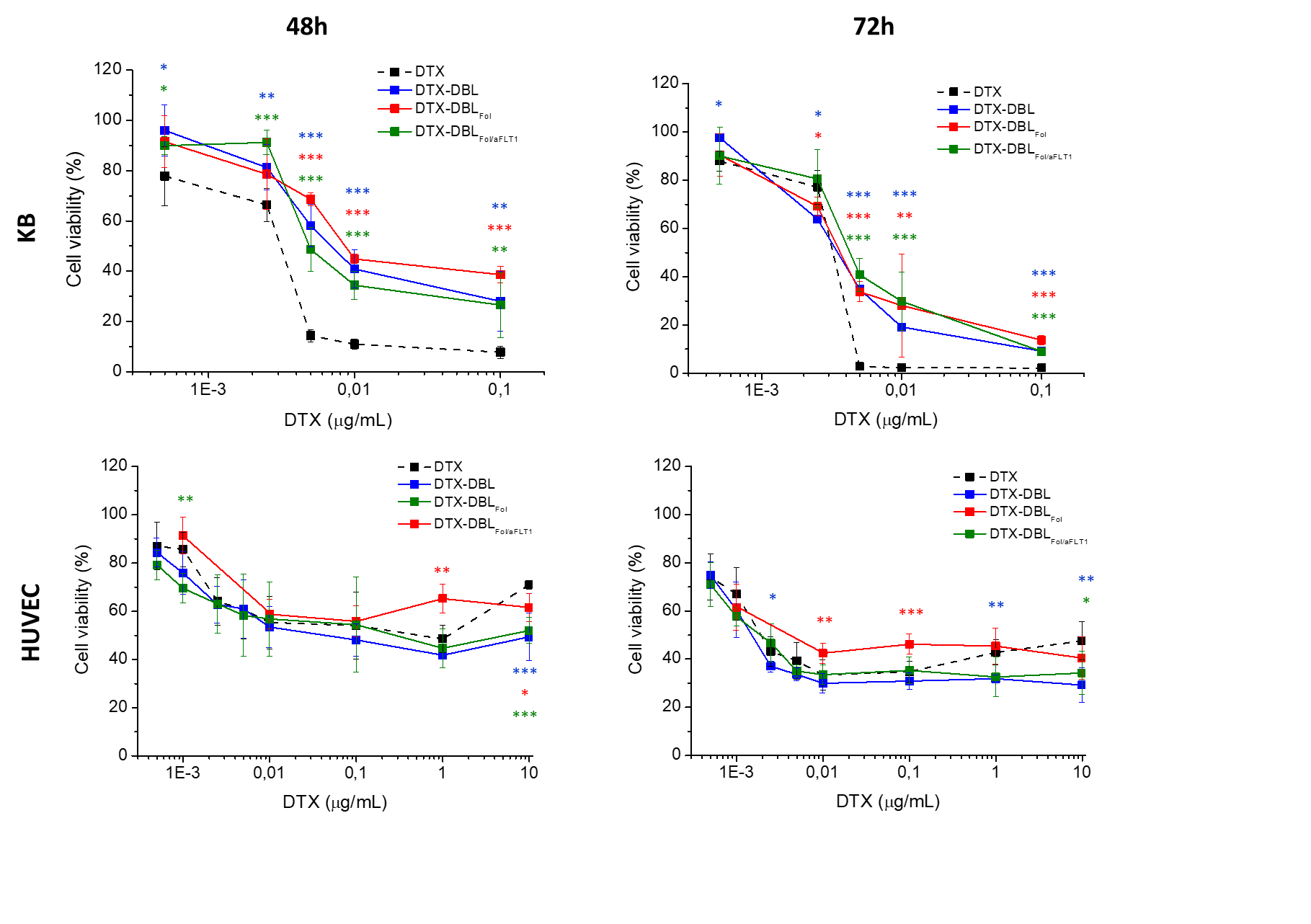


**Figure S2.** Cytotoxicity of nanoparticles on monolayer cell cultures. Cytotoxicity of nanoparticles loaded with Docetaxel (DTX-loaded NPs) *vs.* free DTX in KB and HUVEC cells after 48 h or 72 h of incubation measured through the MTS assay (CellTiter 96^®^ AQueous One Solution Cell Proliferation Assay). DTX dose: 0.001-0.1 μg/mL and 0.001-10 μg/mL for KB and HUVEC cells, respectively. Data are the mean values ± SD of at least three independent experiments carried out in triplicate. *p<0.05, **p<0.01, ***p<0.001 *vs.* free DTX (*Student’s t-test*).


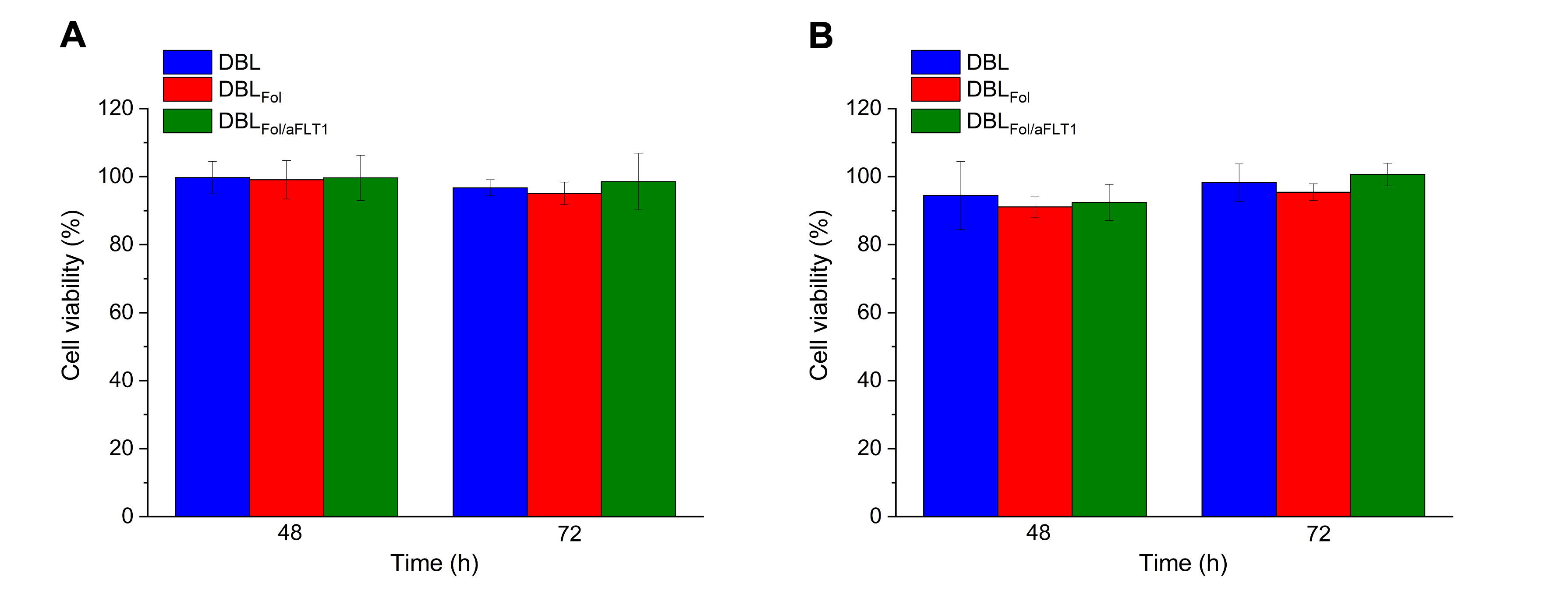


**Figure S3.** Cytotoxicity of empty nanoparticles in KB (A) and HUVEC (B) cell monolayers measured after 48 or 72 h of cell incubation and measured through the MTS assay (CellTiter 96^®^ AQueous One Solution Cell Proliferation Assay). Data are mean values ± SD of at least three independent experiments carried out in triplicate.


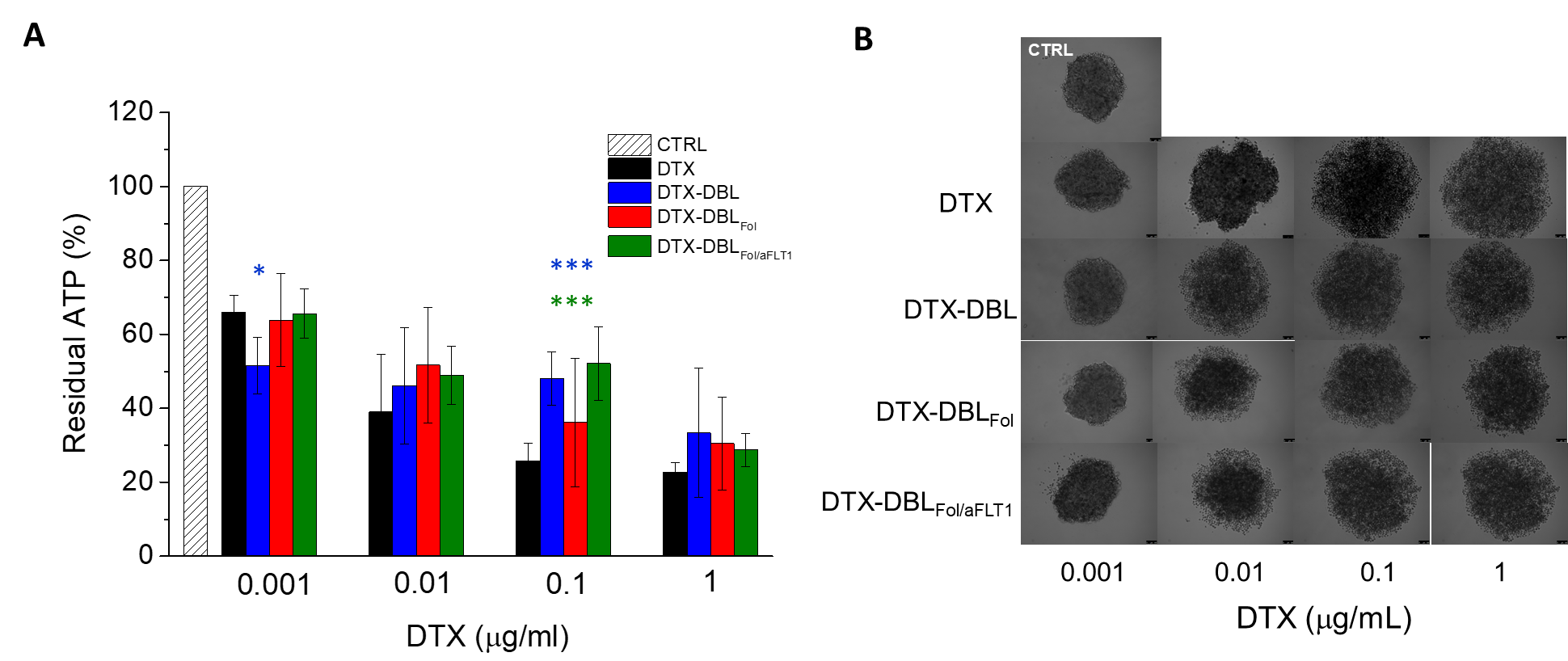


**Figure S4.** Cytotoxicity of nanoparticles loaded with DTX (DTX-loaded NPs) in KB spheroids. A) Percentage of residual ATP in the spheroid measured using the CellTiter-Glo^®^ 3D Cell Viability Assay after 48 h of treatment with DTX-loaded NPs or with the free drug (DTX= [0.001-1 μg/mL]). Data are mean values ± SD of at least three independent experiments carried out in triplicate. *p<0.05, ***p<0.001 *vs* free DTX (*Student’s t-test*); B) Bright-field images of spheroids after 48 h of treatment with DTX-loaded NPs at different doses. Scale bars: 100 μm.


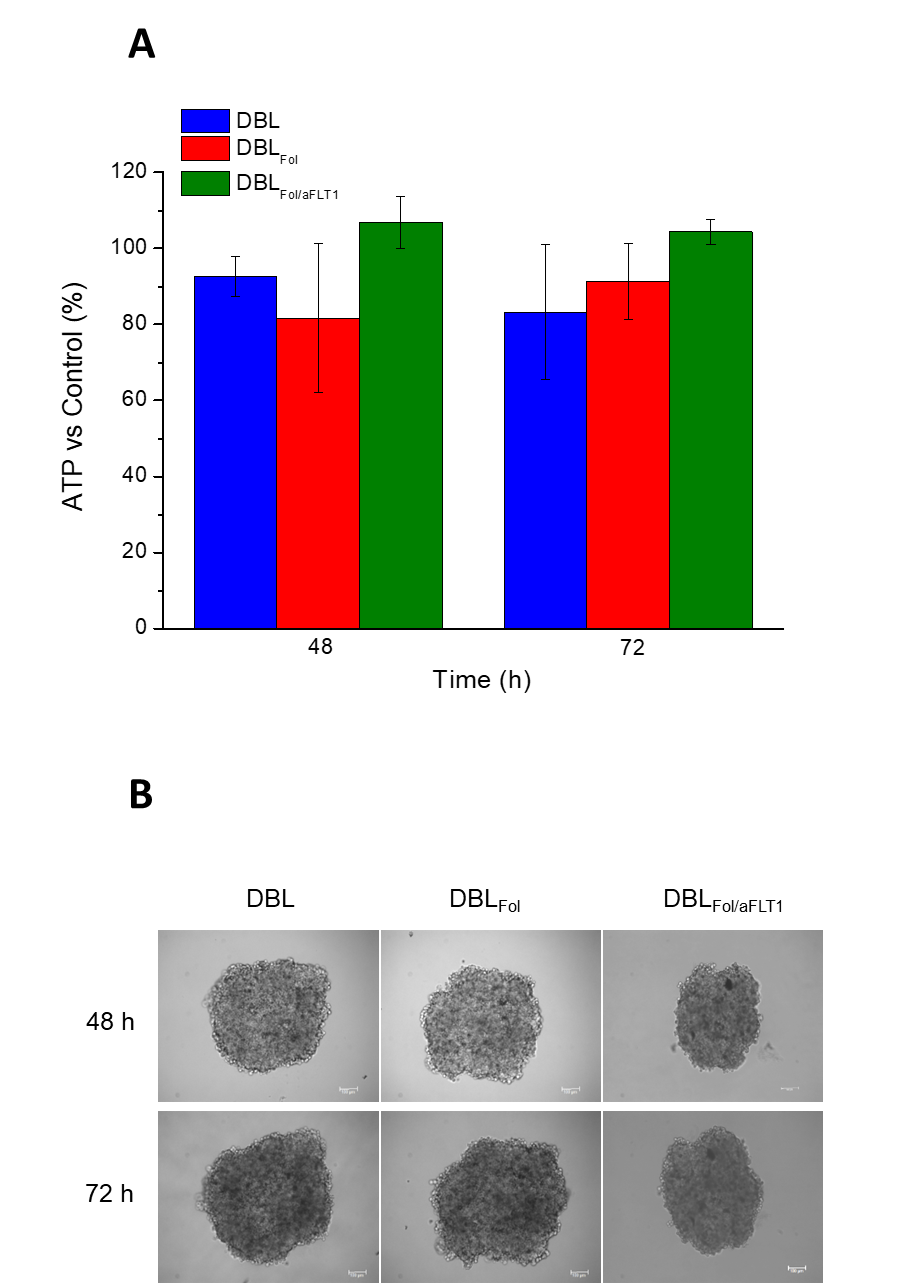


**Figure S5.** Cytotoxicity of unloaded NPs (10 μg/ml) in KB tumor spheroids. A) Percentage of residual ATP in the spheroid measured using the CellTiter-Glo^®^ 3D Cell Viability Assay vs. untreated cells after 48 h or 72 h of treatment. Data are mean values ± SD of at least three independent experiments carried out in triplicate; B) Bright-field images of spheroids after treatment. Scale bars: 100 μm.

**Figure S6**. Toxicity of NPs in non-xenografted zebrafish embryos after 6 days from the treatment with nanoparticles loaded with Docetaxel (DTX-loaded NPs) vs the free drug ([DTX] = 25 ng/animal). Data are mean values ± SD of at least three independent experiments, carried out with at least 25 embryos/experiment.

##


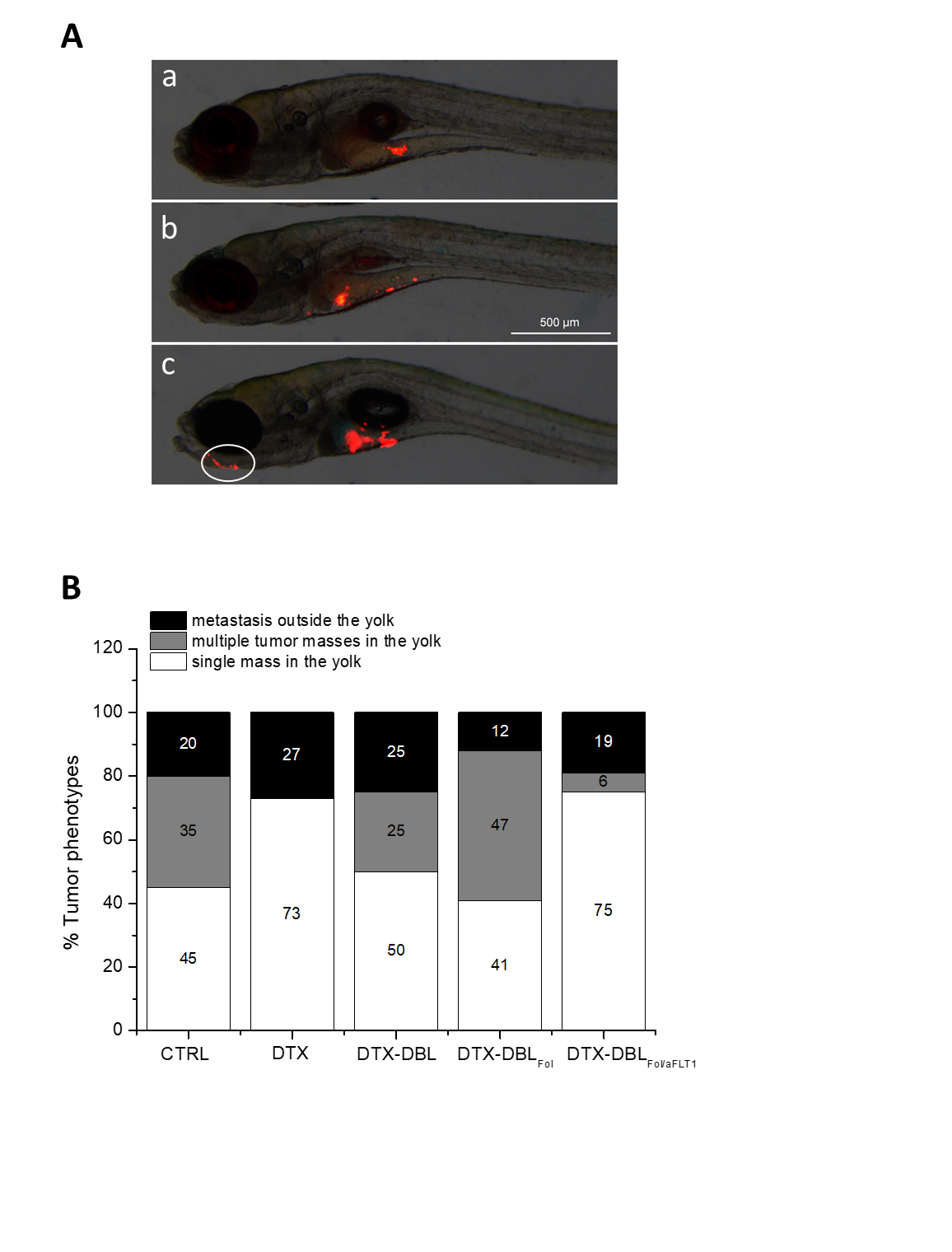


**Figure S7.** A) Images of different tumor phenotypes observed in the embryos: single masses located in the yolk (a), multiple masses located in the yolk (b), tumor masses in the yolk plus metastasis-like masses (white circle) located outside yolk region (c). B) The relative percentages of tumor phenotypes analyzed 6 days-post treatments from fluorescence microscopy images.
